# Axillary lymphadenopathy in a high-risk breast screening patient following the COVID-19 vaccine: a diagnostic conundrum

**DOI:** 10.1259/bjrcr.20210063

**Published:** 2022-03-09

**Authors:** Besma Musaddaq, Adam Brown, Sam Dluzewski, Teresa Marafioti, Anmol Malhotra

**Affiliations:** 1 Department of Radiology, Royal Free Hospital, Royal Free London NHS Trust, London, UK; 2 UCL Cancer Institute, University College London, London, UK

## Abstract

A number of COVID-19 vaccines have been approved worldwide to help tackle the pandemic. As with many vaccines, this causes a reactive axillary lymphadenopathy which can mimic potentially metastatic disease in a breast screening patient. It is therefore important to be aware of this side-effect of the vaccination when evaluating the axilla in a breast screening patient. We present a case of biopsy-proven unilateral reactive axillary lymphadenopathy in a high risk BRCA carrier following administration of the Astra Zeneca vaccine.

## Case presentation

A 57-year-old high-risk BRCA gene carrier patient presented for her annual screening MRI this year. The patient had previously been diagnosed with a left breast cancer 5 years ago for which she had undergone a wide local excision and radiotherapy.

## Investigations

**Figure 1.**
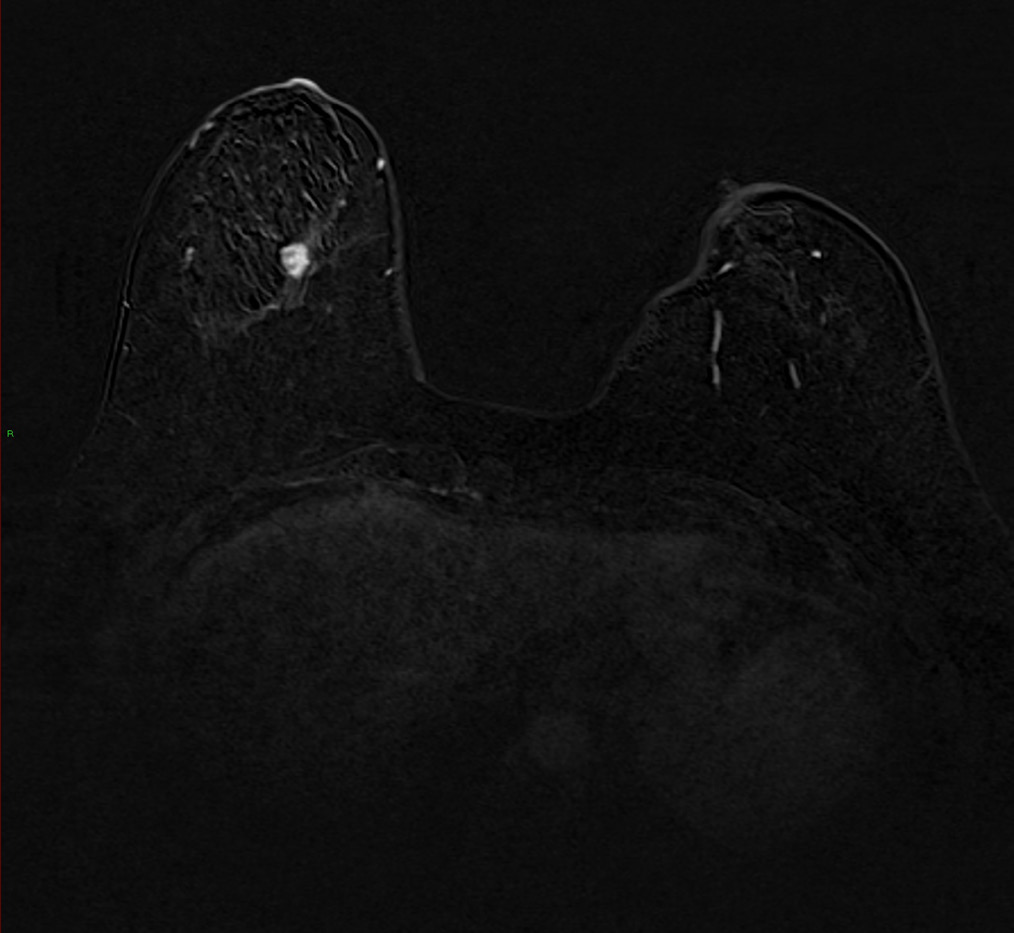
MRI post-contrast subtracted axial image of the breast. There is a 12 × 7 mm lesion within the lower central aspect of the breast. This demonstrated Type 3 enhancement. This was given an MRI grading of MRI 4 (BI-RADS 4B). No abnormal axillary lymph nodes were detected at the time.

On her current screening MRI, a 12 × 7 mm lesion was demonstrated in the lower central aspect of the breast with Type 3 enhancement (Figure 1). This was given an MRI grading of MRI 4 (BI-RADS 4B). No abnormal axillary lymph nodes were detected at the time.

**Figure 2.**
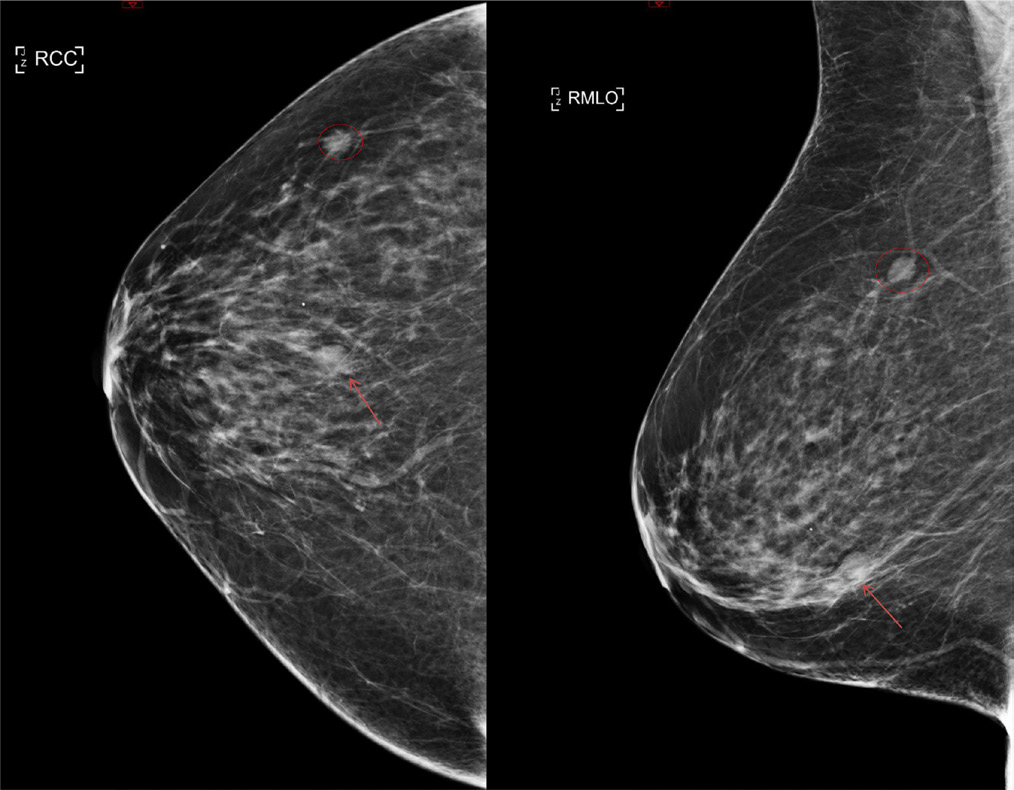
MLO and CC mammogram views of the right breast demonstrate an asymmetric density in the lower central part of the breast measuring 14 mm (arrow). This was given a mammographic grading of M4 (BI-RADS 4B). There is also a long-standing opacity in the upper outer quadrant of the right breast corresponding to the known fibroadenoma (circle). CC, craniocaudal; MLO, mediolateral oblique.

The patient was recalled for further assessment at the screening unit. A screening mammogram was also performed at the same time which showed a new asymmetric density in the lower central part of the breast which did not disperse on further tomosynthesis views (Figure 2). This was given a mammogram grading of M4 (BI-RADS 4B).

**Figure 3.**
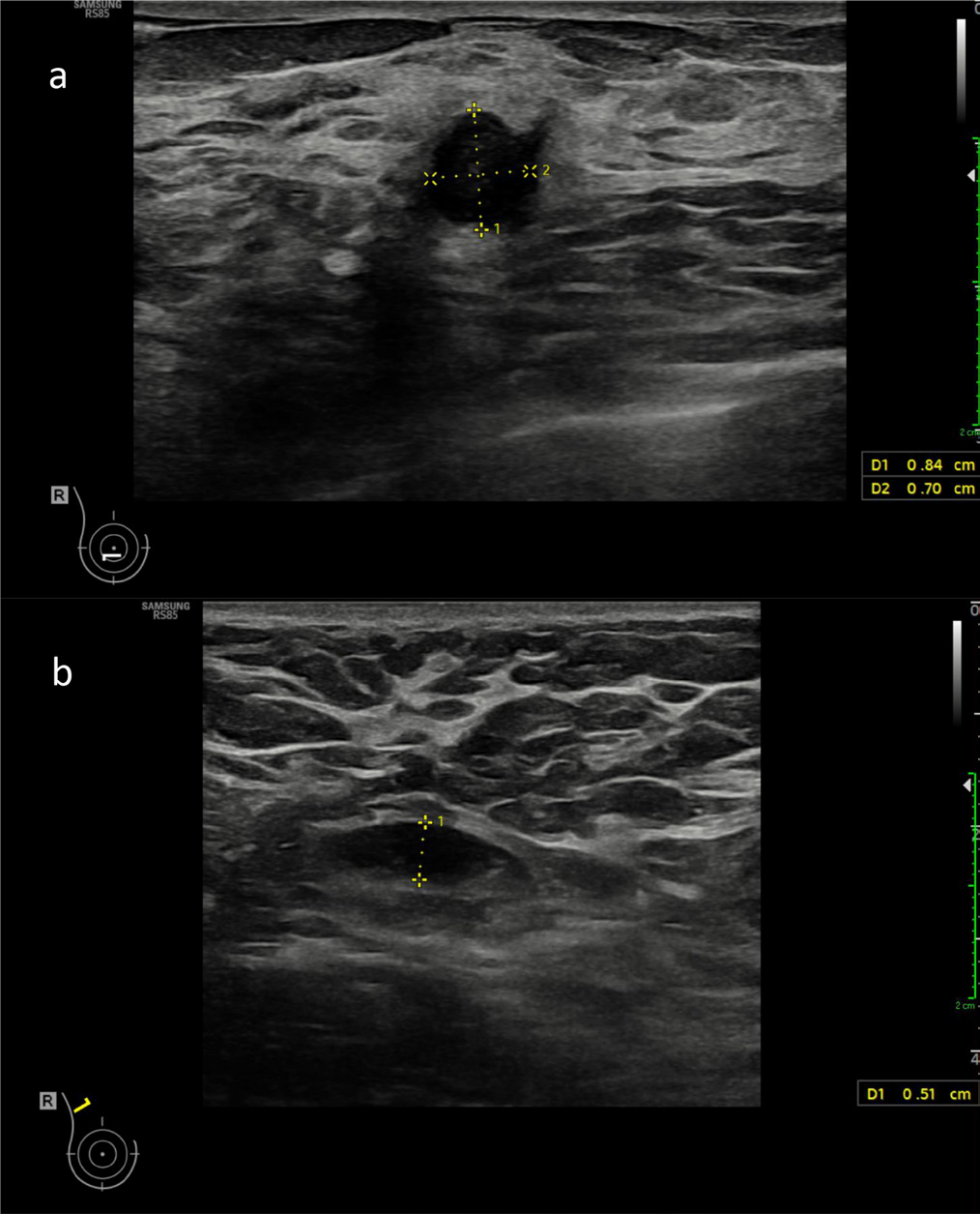
(a) Ultrasound image demonstrating an 8 × 7 mm necrotic hypoechoic irregular contour lesion in the 6A position of the right breast corresponding to the lesion seen on MRI. This was given an ultrasound grading of U5 (BIRADS 4). (b) Ultrasound image showing abnormal right axillary lymph node with increased cortical thickness measuring 5 mm. This was on the same side as the patients COVID-19 vaccine injection.

The ultrasound revealed a vascular 8 × 7 mm necrotic hypoechoic irregular contour lesion in the 6A position of the right breast corresponding to the lesion seen on MRI and the asymmetric density seen on mammography (Figure 3a). This was given an ultrasound grading of U5 (BI-RADS 5). Note was also made of a benign unchanged fibroadenoma in the 3 o’clock position of the right breast. There were abnormal right axillary lymph nodes with increased eccentric hypoechoic cortical thickening measuring up to 5 mm (above the normal value of 2.3 mm used at our institution, Royal Free London NHS Trust) (Figure 3b). The lymph node measured 9 mm in short axis and 19 mm in long axis. There was no increased cortical vascularity. A normal fatty hilum was retained. This had not been seen on previous screening examinations.

The patient revealed that she had received her first dose of the Astra Zeneca Vaccine in the right arm 3 days prior to her attendance to the assessment clinic but after she had had the MRI. Two 14g core biopsies were performed of the suspicious lesion in the lower central part of the right breast and of the abnormal right axillary lymph node.

**Figure 4.**
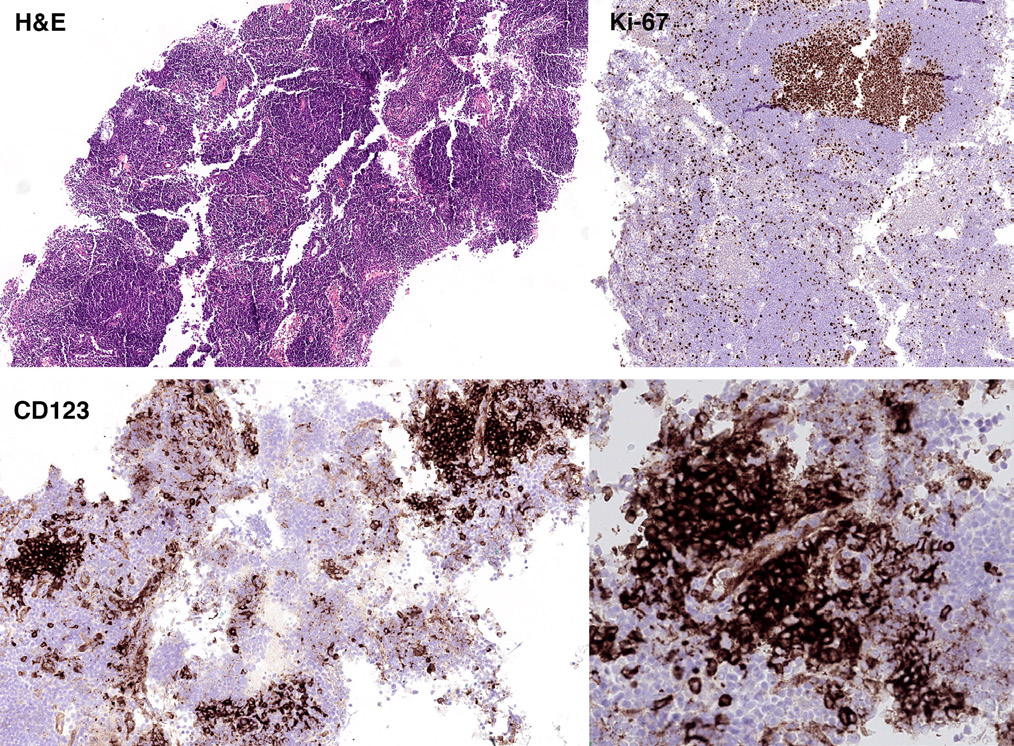
Histology slide of biopsied lymph node. The overall findings were those of reactive lymph node with follicular hyperplasia. No areas of necrosis or granulomata were observed.

The histopathology for the suspicious right breast U5 lesion showed a Grade 2 invasive ductal carcinoma and the right axillary lymph node demonstrated a reactive lymph node with follicular hyperplasia (Figure 4). The normal architecture of the lymph node was preserved. Secondary follicles with reactive germinal centers that were positive for CD20, CD10, BCL-6, and negative for BCL-2 were found. The T cell area was expanded, and it contained small CD3 positive T cells and scattered CD30 positive immunoblasts. One common feature was the presence of clusters of plasmacytoid dendritic cells (PDCs) that had a perivascular localisation and often adjacent to secondary follicles. The PDC were strong positive for CD123 and weak and heterogeneous for BCL-2. The Ki-67 proliferation fraction was high within the germinal centres but not in the PDC clusters. No areas of necrosis or granulomata were observed.

## Differential diagnosis

Given that the patient had a new lesion within the breast and she was a BRCA carrier, there was concern that the axillary lymphadenopathy may represent metastatic disease. Other potential causes include metastatic disease from another primary, infection and inflammation.

## Treatment

The patient is due to undergo a wide local excision for the malignant breast lesion.

## Discussion

As of 8 March 2021, 4 223 232 people have tested positive for COVID-19 in the UK with 140,062 patients succumbing to the virus. 22,377,255 people have received their first dose of the vaccine with 1,142,643 receiving their second doses.^1^ The UK was one of the first counties to approve COVID-19 vaccines with the Pfizer/BioNTech vaccine being approved on 02 December 2020 followed by the AstraZeneca vaccine which was approved on 30 December 2020 and the Moderna vaccine on 08 January 2021.

With the increased administration of the vaccination, there have been a number of cases of clinically or radiologically detected unilateral axillary lymphadenopathy. Ipsilateral axillary swelling and tenderness has been reported in 11.6 and 16.0% of recipients following the first and second dose of the Moderna vaccine respectively.^2^ 0.3% of participants in the Pfizer/BioNTech reported ipsilateral axillary lymphadenopathy versus <0.1% patients in the placebo group.^3^

This can pose diagnostic difficulties when assessing breast screening patients where a reactive lymph node could be mistaken for metastatic disease.

There have been a number of case reports and case series published demonstrating the link between the COVID-19 vaccine and axillary lymphadenopathy. To our knowledge, this is the first case report that demonstrates histopathological proven ipsilateral reactive axillary lymphadenopathy secondary to the Astra Zeneca vaccine in a patient with a malignant breast lesion on the same side. This illustrates the diagnostic difficulty that can occur in such patients when assessing metastatic disease spread.

This issue has been recognised by the Society of Breast Imaging which has issued guidance on how to manage axillary adenopathy in patients who have received the COVID-19 vaccine.^4^ In the UK, the Drug Safety Research Unit has advocated that breast screening appointments should be scheduled to take place before females receive a first dose of COVID-19 vaccine or 4–6 weeks after the second dose when possible.^5^ Gadolinum administration does not help in differentiating reactive lymph nodes from malignant lymph nodes on MRI.

Our case illustrates the diagnostic dilemma posed to clinicians in patients who are at increased risk of breast cancer and present with unilateral axillary lymphadenopathy following COVID-19 vaccine administration. Further guidance and awareness is needed to ensure that patients are managed appropriately and not subjected to unnecessary biopsy and investigation.

## Learning points

1. This case illustrates the difficulty of ascertaining the cause of axillary lymphadenopathy in a high-risk breast screening patient when a patient has received the COVID-19 vaccine as this may be reactive.
2. Axillary lymphadenopathy can occur due to a number of causes including metastatic disease or can be reactive secondary to infection or inflammation.
3. As more patients receive the COVID-19 vaccine, further guidance is required as to how best to manage breast screening patients with axillary lymphadenopathy who have just had the vaccine.
